# Systems biology as an emerging paradigm in transfusion medicine

**DOI:** 10.1186/s12918-018-0558-x

**Published:** 2018-03-07

**Authors:** James T. Yurkovich, Aarash Bordbar, Ólafur E. Sigurjónsson, Bernhard O. Palsson

**Affiliations:** 1 Department Bioengineering, University of California, San Diego, 9500 Gilman Drive, La Jolla, 92093 USA; 2 Bioinformatics and Systems Biology Program, University of California, San Diego, 9500 Gilman Drive, La Jolla, 92093 USA; 3 Sinopia Biosciences, 600 W Broadway Suite 700, San Diego, 92101 USA; 4 School of Science and Engineering, Reykjavík University, Hringbraut 101, Reykjavík, 101 Iceland; 5 The Blood Bank, Landspítali-University Hospital, 9500 Gilman Drive, Reykjavík, 101 Iceland; 6 Department of Pediatrics, University of California, San Diego, 9500 Gilman Drive, La Jolla, 92093 USA

**Keywords:** Systems biology, Red blood cell, Transfusion medicine, Metabolism, Storage lesion

## Abstract

Blood transfusions are an important part of modern medicine, delivering approximately 85 million blood units to patients annually. Recently, the field of transfusion medicine has started to benefit from the “omic” data revolution and corresponding systems biology analytics. The red blood cell is the simplest human cell, making it an accessible starting point for the application of systems biology approaches.

In this review, we discuss how the use of systems biology has led to significant contributions in transfusion medicine, including the identification of three distinct metabolic states that define the baseline decay process of red blood cells during storage. We then describe how a series of perturbations to the standard storage conditions characterized the underlying metabolic phenotypes. Finally, we show how the analysis of high-dimensional data led to the identification of predictive biomarkers.

The transfusion medicine community is in the early stages of a paradigm shift, moving away from the measurement of a handful of chosen variables to embracing systems biology and a cell-scale point of view.

## Background

The human red blood cell (RBC) has long been a starting point for the application of systems biology. The RBC is an ideal model cell because of its relative simplicity and its intrinsic accessibility, resulting in a vast amount of available data. Further, it is of great importance for our understanding of human health and physiology—RBCs account for over 84% of the native cells in the human body by number, making them the most numerous cell type by a large margin [1]. Thus, many of the first whole-cell modeling efforts targeted the RBC. The late 1980s saw the development of the first whole-cell model of the human RBC [2–5]. Since then, other models of the RBC have focused on various aspects of its physiology, from metabolism [6] to its structural properties [7].

Recently, omics technologies have been applied to study RBCs under cold storage for use in transfusion medicine [8]. The transfusion of RBCs has long been an integral part of healthcare [9–11], with approximately 85 million RBC units transfused worldwide annually [12]. RBCs are stored in tightly-regulated, non-physiological conditions (i.e., packed in plastic blood bags in a static environment at 4°C), leading to many changes in the biochemical and physiological properties of RBCs. Over the past several decades, the transfusion medicine community has made great progress in defining a central paradigm that outlines these biochemical and morphological changes—the so-called RBC “storage lesion” (RSL)—that red cells undergo during cold storage [13–16]. Such changes include a decrease in 2,3-diphosphoglycerate (2,3-DPG) levels, an increase in endothelial adherence, and morphological modifications to the shape and structure of the cells. Some of these changes are reversible upon transfusion (e.g., 2,3-DPG levels), while some of the morphological changes can be irreversible.

As omics data characterizing RSL are being generated, the field of transfusion medicine provides opportunities for systems biologists [17]. In particular, metabolomics data have become a central part of the effort to better understand RSL [18]. Profiling the metabolic state of the cell is an important approach that allows a functional interpretation of cellular biochemistry [19]. With the availability of such data, systems biology methods can be applied to study and understand RSL in considerable detail. While correlations are important for the practice of medicine, an actionable and mechanistic understanding of relevant physiological phenomena is desired [20, 21]; systems approaches have already proven valuable through the evaluation of drug therapies [22, 23], identification of biomarkers for cancer [24], and the prediction of oncogenes in cancer conditions [25]. Here, we discuss how the study of RSL is being added to this list.

## Three key ingredients form the path to meaningful multi-omic data integration

**Fig. 1:**
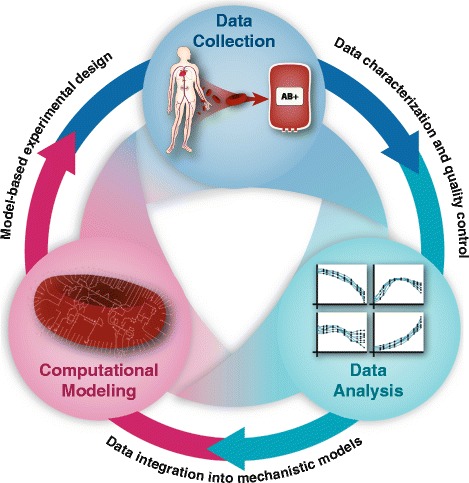
Three key ingredients. Three key ingredients come together to form a workflow capable of extracting knowledge from omics data

There have been a multitude of examples of how a systems biology approach can be applied to study a variety of organisms and biological questions of interest [26–29]. The systems biology approach is an inherently iterative process of refinement that unites three key ingredients: data collection, analysis, and computational modeling (Fig. 1). The first ingredient is data collection. Working in conjunction with blood centers to ensure that standard quality controls are met is vital for generating high-quality data. Absolutely quantified (i.e., using standards to determine concentration values instead of relative amounts) metabolomics data—while more costly—can yield greater benefits since it can be integrated with quantitative, mechanistic models. The data sets described here include time course metabolomics data (both exo- and endo-metabolomic) and other hematological measurements routinely performed in blood banks, such as hemolysis.

Time-resolved metabolomics data have yielded important insights into metabolic physiology, especially when the resolution of the time course captures the time scale of key metabolic changes. For RBCs under storage conditions, these key metabolic changes occur on time scales on the order of days, not weeks (the historical-used time increment). In the experiments discussed below, data is collected every three to four days, providing a more complete characterization of these temporal dynamics. Exploration of various perturbations to the standard storage conditions will help elucidate RBC metabolic properties. Such perturbation experiments might be informed by previous experiments or by computational models [20].

The second ingredient is the application of multivariate data analysis to the large data sets generated. Multivariate statistical analyses can reveal the overall structure of the data sets and trends within the data. In particular, methods like principal component analysis (PCA) [30], partial least squares discriminant analysis (PLS-DA) [31], and independent component analysis (ICA) [32] have been used effectively to analyze complex metabolomics data sets. Care must be taken when choosing a method—statistical methods have specific applications and cannot be blindly applied to raw data; fortunately, there are several excellent resources that provide guidance for this process [30, 33]. Although the data sets generated and analyzed here are large compared to the history of the field, they do not qualify as “Big Data.” In the future, genetic information and other parameters may enrich this data, as has been demonstrated by the generation of personalized RBC models [34]. Notably, the REDS-III initiative—a collaborative, international research program that promises to evaluate 14,000 distinct donors—will directly address hypotheses regarding the effects of genetic variation on donor-specific RSL properties such as hemolysis [35]. The availability of such rich data will provide great potential for systems analyses.

The third ingredient is a computational, mechanistic metabolic network model capable of integrating disparate data types. Such models incorporate the results of statistical analyses to generate biological insights and testable hypotheses. A metabolic network specific to the RBC has been generated by mapping multiple proteomic data sets onto the reconstruction of the global human metabolic network [36, 37]. This mapping has resulted in a functional metabolic network of the RBC containing 283 metabolic genes [6] and includes contiguous known pathways. The specifics of this network have been further delineated through a comprehensive manual curation of the literature (the “bibliome”). This reconstructed metabolic network inherently includes available information about the metabolome, proteome, and bibliome [38], truly representing multi-omic data integration.

Several years ago, the use of systems biology principles in the transfusion medicine field were proposed as a way to extend the lifetime of stored RBC units [17]. Since then, the principles outlined above have been used to study RBC storage from a systems perspective. Here, we review the outcome of several of these efforts and try to contextualize these results.

## Multivariate statistical analysis reveals a three-phase metabolic decay

The first major step in a systems biology characterization of the storage process was to understand the baseline RBC metabolic behavior during storage [39]. RBC units were collected from 20 individuals and stored in saline-adenine-glucose-mannitol (SAGM) media. SAGM media is used throughout Europe, the United Kingdom, Australia, Canada, and New Zealand, although it is not licensed by the FDA [40]. Over 140 metabolites and hematological variables (e.g., hematocrit, pH) were absolutely quantified at 14 time points over 45 days of storage, three days past the FDA-regulated maximum shelf life of 42 days. With measurements taken every three to four days, the resulting time resolution was finer than historically practiced in the transfusion medicine field (that typically uses weekly sampling) and allowed for the capture of previously unobserved behaviors. The quantitative nature of the measurements is important for use in mechanistic models [41] while further allowing for an assessment of whether certain compounds reached toxic levels, thereby enabling the corroboration of previous results for the accumulation of compounds such as hypoxanthine [42, 43].

Initial global characterization of the data was achieved using PCA of the raw metabolomics data, revealing two distinct metabolic shifts that occur during the 42 day shelf-life of a stored RBC unit. These shifts in metabolic state—occurring at days 10 and 17—showed that RBCs do not undergo a simple linear decay process but instead go through a defined series of three metabolic states each with distinct characteristics. A similar three-phase metabolic decay profile was being observed in parallel in independent studies and in different storage media [44] and has since been shown in other independent studies [45]. Further, measuring endothelial biomarkers such as hcDNA levels helped determine that blood transfused from the different states had differing effects on the endothelium [39]. These results showed that RBC units transfused from the third state resulted in endothelial tissue damage.

The previously reported high concentration of 2,3-DPG that depletes over time was observed, as well as the initial increase and subsequent decrease in ATP levels after the first shift at day 10. It was observed that the “metabolic inflection points” (i.e., the points in time at which the metabolic shifts in the PCA plots occur) approximately coincide with the depletion of extracellular adenine and onset of accumulation of hypoxanthine and xanthine in the storage medium. One notable novel observation from the quantitative metabolomics data was the existence of a large intracellular malate pool (greater than 1 mM). The discovery of these metabolic phases and their characterization was only possible through the use of a systems-level perspective.

## Mechanistic models informed by quantitative data generates hypotheses and understanding

The multi-variate data analysis results provided by this baseline study of RBC metabolic decay have proven to be a useful starting point for mechanistic model-based analysis. Since the baseline metabolomics data are absolutely quantified, they can be integrated into mechanistic, cell-scale models capable of making quantitative predictions [46, 47]. These models predicted that the large 2,3-DPG pool is utilized to generate ATP using 2,3-DPG as a proton buffer through the reversal of bisphosphoglycerate mutase. The catabolism of 1,3-DPG generates two ATP, while the expected dephosphorylation of 2,3-DPG to 3PG generates only one ATP. Given that the initial 2,3-DPG pool is high, the shift in degradation route has a large influence on the overall ATP generation during storage. While an interesting prediction, thermodynamics might suggest that this behavior is unlikely to occur, highlighting a need for additional study. Others have built on these modeling predictions by providing an examination of the potential metabolic blockade in glycolysis at glyceraldehyde 3-phosphate dehydrogenase (GAPDH) based on redox proteomics analyses and ^13^C _1,2,3_-glucose tracing to elucidate this mechanism [48].

The quantitative model also made predictions about the metabolic fate of citrate, a compound added to the storage medium as an anticoagulant during blood collection. These results were validated [47] and then further explored in a follow-up study in which labeled glucose, citrate, and glutamine were added to RBC units [49]. More recent efforts have examined how this mechanism is impacted by oxygen saturation in stored RBCs [50]. Together, these studies suggest that RBCs can metabolize carbon sources such as citrate to produce lactate and glutamate. These examples illustrate how quantitative metabolomics data enables model-based analysis that leads to a biochemically-informed mechanistic understanding of metabolic changes that unfold in the RBC during storage.

## Perturbing the storage conditions

**Fig. 2:**
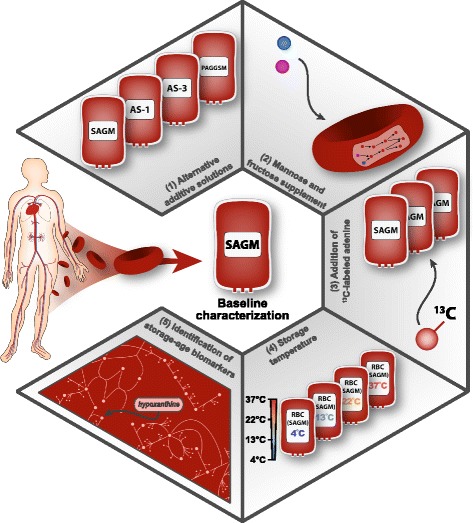
Characterizing RBC storage conditions. Baseline characterization and perturbation experiments on stored RBCs. Perturbation experiments examined the effect of (1) alternative media formulations, (2) supplementation with additional sugars, (3) addition of adenine to the media, and (4) a change in storage temperature. An additional set of experiments (5) yielded a robust set of storage-age biomarkers

Having characterized the baseline metabolic behavior of stored RBCs, the next step in the systems biology characterization was to determine whether the storage conditions could be perturbed in such a way that this three-phase metabolic decay process was affected. Four perturbations representing pressing questions were identified and examined (Fig. 2): (1) does the three-phase decay pattern manifest itself only in SAGM media, or is it present in other storage media types used in transfusion medicine?; (2) do alternative sugars support ATP levels better than glucose?; (3) is the depletion of adenine the cause of the metabolic shifts?; and (4) how does storage temperature affect the metabolic network?

### (1) Does the storage media affect the metabolic decay pattern?

With the baseline behavior in SAGM media characterized, it was important to determine whether RBCs stored in other additive solutions exhibited similar metabolic behavior [51]. RBC units from 12 individuals were stored in SAGM [52, 53], AS-1 [54], AS-3 [54, 55], and PAGGSM [56] for 45 days; an excellent discussion of the differences among these solutions can be found in [40]. Samples were collected and metabolically profiled at 14 time points during storage. These media types were chosen because they represent the most widely-used additive solutions in Europe (SAGM, PAGGSM) and the United States (AS-1, AS-3) [40].

Several changes in basic metabolic behavior were observed across the four additive solutions. Notably, citrate uptake and metabolism was increased in AS-3 and PAGGSM compared to that of SAGM and AS-1. Corresponding changes in intracellular malate concentrations suggest that citrate uptake impacts malate utilization. Labeled citrate added to the bag prior to storage in SAGM showed that citrate is taken up and converted to intracellular malate, contributing to the large pool previously observed in the baseline characterization. This behavior has been shown to occur in other media [49] and was computationally predicted and validated in the baseline data [47]. Statistical analyses indicated that the difference in citrate uptake and metabolism impacted glycolytic function, with small differences noted in glycolytic flux among the additive solutions. Ultimately, while there were some minor differences among the four solutions, the conclusion was that the overall three-phase pattern of metabolic decay is the same in alternate storage solutions.

### (2) Do alternative sugars support ATP levels better than glucose?

The baseline data showed that fructose and mannose found in the plasma collected with the RBCs from the donor are rapidly metabolized and depleted during the first metabolic phase. Mannose and fructose have been shown to be metabolized through different pathways than glucose in RBCs [57, 58], thus providing potential benefit over glucose as the primary energy source for metabolism. Further, the potential positive and negative effects of fructose are not yet clearly understood [59, 60], making it a good target for further study. Following in the footsteps of work by Beutler and Duron [61] and by Dawson and colleagues [57, 62], RBC units were supplemented with mannose and fructose to better characterize alternate sugar metabolism during storage [63]. These units were metabolically profiled at 14 time points over 25 days of storage in SAGM media.

These experiments suggested that the metabolism of mannose and fructose at 4°C reflects their metabolism at 37°C, but this is a potentially misleading result due to the presence of glucose (i.e., mannose and fructose were supplemented instead of replacing the normal glucose). The timing of the metabolic inflection points was altered slightly with the supplemented sugars, with the observed changes primarily centered in glycolysis. The hypothesized protective effect of fructose was not observed. The additives failed to maintain ATP and 2,3-DPG levels under the tested experimental conditions, although this was likely due to the presence of glucose.

While fructose is known to be taken up through the GLUT5 transporter [64], the exact transport mechanism for mannose uptake has yet to be clearly elucidated. However, it is believed that mannose is transported into the cell via the GLUT1 transporter [58]. GLUT1 is also used by glucose, leading to competition for uptake of the two compounds. The ^13^C labeling results from this perturbation study support the hypothesis that mannose is taken up by GLUT1. More importantly, these results imply that mannose is preferentially taken up and oxidized over glucose. Although some differences in the metabolic state were observed with the addition of mannose and fructose, there is no clear advantage to these supplementations. Ultimately, a better characterization of the metabolism of these sugars could be obtained by replacing glucose with mannose and/or fructose (instead of supplementing) and examining the resulting metabolomics measurements.

### (3) Is the depletion of adenine the cause of the metabolic shifts?

Following the identification of the three-phase metabolic decay observed in SAGM [39], it was observed that the depletion of adenine coincided with the metabolic inflection points observed in the PCA plots. It was hypothesized that these metabolic shifts were due in part to the depletion of adenine. To test this hypothesis, adenine was labeled in both normal and double concentrations in SAGM media, and metabolomics measurements were made at 10 time points over 31 days of storage [65].

It was observed that the RBCs consumed approximately 1.5 mg/L adenine per day over the first eight days of storage, almost depleting the total adenine concentration in the bag toward the end of the first metabolic phase. During this first phase, adenine was converted into inosine and IMP but not ATP. By day 18 of storage (the end of the second phase), the extracellular adenine was completely depleted.

Having detailed adenine metabolism under standard storage conditions, its initial concentration was doubled to see if its depletion was the cause for a metabolic shift. The identical consumption rate of adenine was observed until day 18, at which point adenine was no longer taken up by the RBCs. In other words, it appears that the perfect amount of adenine is found in SAGM media; adding any more would result in adenine sitting in the extracellular media without being consumed by the RBCs. One possible explanation for this result is that this behavior was previously characterized during the development of SAGM media but never published (and has now been re-discovered years later). One notable observation was that the higher levels of adenine resulted in a buildup of 5-methylthioadenosine. The conclusion of this study was that adenine is not responsible for the observed metabolic shifts: there is another internal process that leads to the cessation of adenine uptake.

### (4) Does the storage temperature affect the metabolic network?

The previous studies all examined perturbations to the storage media itself. One important aspect of the storage conditions, however, is the low temperature at which RBC units are stored (4°C) and how this low-temperature environment affects RBC metabolism. In order to investigate these effects, RBC units were stored at 4°C, 13°C, 22°C, and 37°C—temperatures that span the *ex vivo* (storage) and in vivo (body) temperatures—and metabolically profiled [66]. Bags were stored for 21 days (4°C, 13°C, and 22°C) and 7 days (37°C) because the metabolic shifts were anticipated to occur earlier at higher temperatures.

This study investigated whether the three-phase decay pattern was observed at all temperatures. PCA was performed on the metabolomics measurements, observing that these trends were preserved but accelerated with increasing temperature. The temperature dependences for individual metabolites and reaction fluxes were calculated, finding that the response of these individual network components did not scale uniformly with increasing temperature. In particular, the behavior of the ions was quite different at low temperatures due to the known inactivation of the sodium/potassium pump [67, 68]. Together, these results showed that the RBC metabolic network is robust against the accumulation or depletion of intermediate metabolites.

These results open the door for another interesting possibility: can the temperature dependences calculated here be used to run high throughput screens of additional perturbation experiments at higher temperatures? Our results suggest that an RBC unit stored at a temperature of only 13°C would only need to be stored for approximately 14 days to observe the equivalent 42 day storage behaviors observed at 4°C. Since the global network-level changes were consistent at higher temperatures, any screens in which the three-phase decay pattern was disrupted could then be investigated in detail under the proper conditions. It is important to note, however, that there is still further evaluation required; the temperature-driven effects on ion homeostasis—which activates ion-dependent cascades (e.g., calcium-induced eryptosis, a phenomenon known to occur during prolonged storage [69, 70])—would not be taken into account by such measurements. If these considerations were properly addressed, a shorter experimental time would not only allow for high throughput screens, but it also yields the very practical consequence of reducing experimental costs. Experiments could be further accelerated if we could find biomarkers that characterize the three-phase decay without the need for full and expensive metabolomic data generation.

## Can we define a set of biomarkers that represent RBC metabolic health?

One obstacle to the routine use of metabolomics data is the high cost of generating them. With the increasing amount of metabolomics data already available for RBCs under storage conditions [18] and relative invariance of the metabolome composition during decay, it is logical to ask if we can identify biomarkers that describe the three-phase decay process. With the large amounts of data available in the literature and the above characterizations of perturbed conditions, have we reached a point where there is a critical mass of data available for true systems analysis leading to the identification of robust biomarkers?

Paglia et al. [71] set out to identify a set of metabolites that could define the three metabolic phases based on normal SAGM decay. A small number of extracellular metabolites were identified because of ease, cost, and reliability of such measurements. Through statistical analysis of existing data sets, eight extracellular metabolites (adenine, hypoxanthine, glucose, lactate, malate, nicotinamide, 5-oxoproline, and xanthine) were identified that can differentiate between the three metabolic states. These “storage-age” biomarkers robustly represent the RBC metabolome throughout the storage process. Initially identified in SAGM media, these storage-age biomarkers were validated in AS-3 and independently verified in a separate laboratory with a different analytical setup and different sample sets [71].

Glucose, lactate, 5-oxoproline, and adenine represent the primary metabolic inputs and outputs can effectively serve as “clocks” for storage time. Further, the large malate pool is related to a major component in the citrate buffer used during processing. The potentially more interesting biomarkers are nicotinamide, hypoxanthine, and xanthine that are directly indicative of the metabolic state. Nicotinamide is one of the components of major cofactors (NAD ^+^/NADH and NADP ^+^/NADPH) and is released from RBCs after approximately ten days of storage. The toxic effects of hypoxanthine and xanthine are well known [72], making them good targets for additional study. Preliminary findings have already shown that hypoxanthine levels correlate with post-transfusion recovery in vivo and are related to purine oxidation and salvage [73, 74], suggesting that hypoxanthine could be a biomarker for more than just storage age.

The utility of these storage-age biomarkers has been shown to transcend their ability to differentiate the three metabolic phases. A recent study showed that the concentrations of these extracellular metabolites at a particular time point can be used to quantitatively predict the concentration of other metabolites in the network [75]. Additional follow up work demonstrated that certain combinations of these biomarkers (based on their location within the metabolic network) could be used to forecast the future values of other metabolites in the network [76]. The identification of these robust biomarkers is important from a practical standpoint: they reflect the fact that the metabolic decay process is fairly invariant under the conditions examined, thus revealing the inherently low dimensionality of the dynamics of the metabolic decay process.

This particular set of storage-age biomarkers needs further validation, not only in additional storage media but also to determine whether they are indicative of in vivo behavior. Other biomarkers have also been suggested that may be able to measure the quality of stored RBCs, such as peroxiredoxin 2 levels [77]. The identification of a robust set of biomarkers that can be used to define both ex vivo (i.e., storage) and in vivo health of RBCs is an area where systems biologists can provide a meaningful contribution to the field of transfusion medicine.

## Toward broader applications of systems biology to transfusion medicine

Transfusion medicine is a major part of healthcare. The results and insights gained from the application of omics data sets and systems biology analytics to stored RBCs will continue to grow in scope and sophistication. As this systems view expands to include additional types of information and data, phenomena such as genetic variation in the human population is likely to come into focus. The community has built large, collaborative efforts like the REDS-III initiative [35] that directly address some of these issues.

The RBC has been and will continue to be a useful model system for applying and developing systems biology approaches. It is the simplest of human cells, easily accessible, and easy to lyse and characterize biochemically. Multi-scale analysis of RBC functions is needed to elucidate its role in human physiology, a fact easily demonstrated by looking at the physiologically-relevant RBC time scales: one second for capillary transit, one minute for average circulatory time, 45 min for ATP turnover, 24 hours for circadian rhythms, and 60 days for its half-life in circulation. It is fair to say that if the systems biology community cannot fully characterize the RBC, the prospects of doing so for more complicated human cell types are dim. Once we succeed with a refined definition of RBC systems biology, it is logical to proceed to the next simplest cell—the human platelet. The presence of organelles (e.g., mitochondria) and signaling pathways will provide challenges beyond those faced in RBC physiology.

The human RBC is not only the ideal target for systems analysis, but it also represents a system of high interest for studying human physiology and is central to transfusion medicine. The RBC is the cell type most amenable to systems analysis through the integration of multiple omic data types into a mechanistic model. These data sets can be gathered to reflect various criteria such as gender, age, and ethnic diversity. Ultimately, there is great promise for the use of systems biology approaches to design experiments informed by a mechanistic understanding of RBC physiology [20]. The field of transfusion medicine therefore holds great opportunity as a place for the development and practical application of systems biology approaches to human physiology and the delivery of healthcare.
